# Supraclavicular skin temperature and BAT activity in lean healthy adults

**DOI:** 10.1007/s12576-015-0398-z

**Published:** 2015-09-29

**Authors:** Anouk A. J. J. van der Lans, Maarten J. Vosselman, Mark J. W. Hanssen, Boudewijn Brans, Wouter D. van Marken Lichtenbelt

**Affiliations:** 1 Department of Human Biology, NUTRIM School for Nutrition, Toxicology and Metabolism, PO BOX 616, 6200 Maastricht, The Netherlands; 2 Department of Nuclear Medicine, Maastricht University Medical Center+ (MUMC+), P Debyelaan 25, 6229 Maastricht, The Netherlands

**Keywords:** Supraclavicular skin temperature, BAT activity, Human adults, iButtons, PET/CT-imaging

## Abstract

The ‘gold standard’ for measuring brown adipose tissue (BAT) in humans is [^18^F]FDG-PET/CT-imaging. With this technique subjects are exposed to ionizing radiation and are therefore limited in the number of scans that can be performed. We investigated the relation between supraclavicular skin temperatures and BAT activity values using a strictly temperature-controlled air-cooling protocol. Data of 36 male subjects was analyzed. BAT activity was evaluated by [^18^F]FDG-PET/CT-imaging and skin temperature was measured by means of wireless temperature sensors. Supraclavicular skin temperature dropped less compared to skin temperatures at other sites (all *P* values <0.01). A significant positive correlation was found between the change in supraclavicular skin temperature with BAT activity (*R* ^2^ 0.23), and the change in supraclavicular skin temperature and non-shivering thermogenesis (*R* ^2^ 0.18, both *P* values <0.01). The correlations indicate that supraclavicular skin temperature (changes) can potentially be used as a qualitative measure of BAT activity and BAT thermogenesis.

## Introduction

Mammals are homeotherms, which mean that they are able to maintain stable internal body temperatures by physiological means [1]. During cold exposure, internal body temperatures can be maintained via two mechanisms; by decreasing heat loss to the environment via vasoconstriction and by increasing heat production [2]. The extra heat can be produced by shivering thermogenesis or by non-shivering thermogenesis (NST). It has been known for many years that BAT is the main site for NST in small mammals and human infants [3, 4]. In rodents, the largest BAT depot is located in between the scapula (i.e., interscapular BAT, iBAT); other BAT depots are the cervical, axillary, mediastinic, and perirenal depots [5]. BAT contains many mitochondria with uncoupling protein-1 (UCP1). This protein uncouples ATP production from the respiratory chain and consequently heat is generated [3, 6]. Besides the abundance of UCP1, BAT is densely innervated by the sympathetic nervous system and is highly vascularized [3, 6]. These characteristics are all in support for BAT’s main function: heat production to maintain core body temperature.

The occurrence of BAT in human adults was known for many years [7, 8]. The findings of Huttunen et al. [9], who showed that outdoor workers have a higher occurrence of BAT compared to control subjects, indicated that cold exposure might be important. It took until 2009 before a direct causal relationship between active BAT and cold exposure was found [10–12]. Additionally, several studies show that cold-induced BAT activity and NST are related [13–17]. This suggests that BAT is also important in maintaining core body temperatures in human adults. Cold-activated BAT in humans is mainly found in the neck, acromial-clavicular, supraclavicular, para-aortic, axillary, paravertebral, and perirenal depots [5, 18]. In order to localize BAT in human adults 2-deoxy-2-[^18^F]fluoro-d-glucose-positron-emission-tomography and computed-tomography ([^18^F]FDG-PET/CT) is commonly used [19]. This scanning technique involves ionizing radiation both by the CT-scan and the administration of a radioactive isotope ([^18^F]FDG) for PET-imaging, and is, therefore, limited in the number of scans that can be performed. Therefore, additional techniques are needed to study BAT without radiation penalty.

From that perspective, measuring BAT temperature could be a qualitative method to verify whether BAT is activated or not, and this could possibly be an indicator of BAT presence. Interscapular BAT can be measured directly in rodents by means of thermistors placed on the interscapular BAT depot [20]. However, this technique is invasive and, therefore, not suitable for employment in humans. Instead, measuring the temperature of the skin overlaying the BAT depots could be an attractive alternative. Indeed, by means of infrared thermography, studies in rodents showed a smaller decrease in interscapular skin temperature upon cooling, compared to tail and back skin temperature [21]. Moreover, skin temperature overlaying BAT-sites is significantly higher than nonBAT-sites [22]. In human adults similar results were found. Measured by means of infrared thermography, supraclavicular skin temperature decreased less (BAT-site) than skin temperature at mediastinum (nonBAT-site) after cold exposure (17 °C for 30 min) [23]. Besides infrared thermography, skin thermistors are frequently used to measure (changes in) skin temperature [24–26]. Skin thermistors are small, relatively cheap, and easy to use.

The studies published so far used different means of cold exposure to activate BAT [24–26]; both water-cooling and air-cooling protocols were used. Besides that, both fixed and individualized cooling protocols were applied. These different protocols can lead to different relations between skin temperatures and BAT activity values [18]. Therefore, in this study we investigated the relation between supraclavicular skin temperatures and BAT activity in human adults during strictly temperature-controlled cold stimulation.

## Materials and methods

### Subjects

In order to study the correlation between skin temperatures and BAT activity, we studied the data of 36 male subjects undergoing an individualized cooling protocol by means of air-cooling. All were healthy, lean, young adults (see Table 1 for subject characteristics). Females were not included due to hormonal effects on thermoregulatory responses [27, 28]. Exclusion criteria were diabetes mellitus, use of beta-blockers, and a history of cardiovascular diseases and asthma or other pulmonary obstructive disease.

**Table 1:** Subject characteristics

Characteristic	
Age (years)	23.4 ± 3.6
Height (m)	1.83 ± 0.1
Weight (kg)	73.5 ± 7.3
BMI (kg/m^2^)	21.9 ± 1.75
Fat mass (%)	16.3 ± 3.9
BAT+/BAT−	34/2

Values are expressed as mean ± SD

BAT+ number of BAT-positive subjects upon cold stimulation

BAT− number of BAT-negative subject upon cold stimulation

### Study design

Subjects were studied in the morning after an overnight fasting period. Subjects swallowed a telemetric pill (CorTemp HT150002; HQ Inc., USA) for core temperature measurements and iButtons (Maxim Integrated Products, USA) were placed for skin temperature measurements. A cannula was inserted in the antecubital vein for [^18^F]FDG administration. Energy expenditure was measured continuously by means of a ventilated hood system (Omnical; Jaeger, the Netherlands). Measurements started with a thermoneutral period of 45 min, and after this the individualized cooling protocol started. After 30 min of stable non-shivering condition the tracer was injected and mild cold stimulation remained for 60 min. Hereafter, all measurements were stopped and subjects were transported towards the scanner for PET/CT-imaging.

### Skin temperatures

**Fig. 1:**
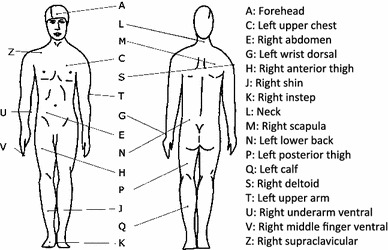
Visualization of the placement of iButtons on 17 sites for skin temperature measurements

On 14 ISO-defined [29] sites iButtons were placed, three additional iButtons were placed at the underarm (ventral; middle in between base of the thumb and elbow) and fingertip (ventral side of middle finger) and above the right clavicular (supraclavicular; BAT location) (Fig. 1). Skin temperatures were measured by means of wireless temperature sensors (iButtons) with a sampling rate of 1 min. The sensors were attached to the skin using Fixomull tape (BSN, Hamburg, Germany). Validation studies showed that means accuracy is −0.09 °C with a precision of 0.05 °C. [29]. Mean skin temperature was calculated as the average temperature of sites A–T (Fig. 1), proximal temperature as the average of chest (C), abdomen (E), scapula (M) and lower back (N), distal temperature as the average of wrist (G) and foot (K). To gain more insight into body insulation, temperature gradients were calculated between core and mean skin, core and distal, and between proximal and distal temperature.

### Individualized cooling protocol by means of air-cooling

Subjects were lying semisupine on a nephrodialysis chair wearing sweatpants, a T-shirt, and socks or on an air-permeable stretcher dressed in shorts and T-shirts. The chair or stretcher was placed in a specially equipped air-permeable tent (Colorado, Altitude Training) in which ambient temperature could be tightly controlled. Average relative humidity during the entire protocol was 40.6 ± 7.6 %. Measurements started with a thermoneutral period of 60 min (average tent temperature 25.9 ± 1.6 °C), followed by an individualized cooling protocol. For this purpose subjects were gradually cooled down until the onset of shivering (tent temperature was lowered on average with 10.5 ± 2.3 °C). After this, environmental temperature was raised by 1–2 °C in order to stop shivering. This was confirmed by EMG measurements and the absence of shivering was checked visually. After 30 min of a stable non-shivering thermogenesis condition (average tent temperature 15.8 ± 1.9 °C), [^18^F]FDG was injected and mild cold exposure remained for 60 min. Using this protocol, BAT activity is studied during maximal non-shivering thermogenesis. After the cooling protocol, subjects were transferred to the PET/CT scanner (Gemini TF PET-CT, Philips).

### PET/CT-scanning protocol

One hour after [^18^F]FDG injection, PET/CT-imaging was conducted. Imaging started with a low-dose CT scan (120 kV, 30 mAs), immediately followed by a PET scan. A total of six to seven bed positions were necessary to cover the area where BAT is usually found. The PET image was used to determine the [^18^F]FDG uptake, and the CT-image was used for PET attenuation correction and localization of the [^18^F]FDG uptake sites. The voxel size of reconstructed PET and CT image sets were 4 × 4 × 4 mm^3^ and 1.172 × 1.172 × 1.172 mm^3^, respectively.

### PET analysis

The scans were analyzed using PMOD software (version 3.0 PMOD Technologies, Zurich, Switzerland). The researcher (MJV) and an experienced nuclear medicine physician (BB) interpreted the PET/CT-images. BAT activity is defined as glucose uptake in fat tissue. This is expressed in mean standardized uptake value (SUV mean), with maximal SUV as the maximum value in that region. The regions of interest were manually outlined in each slide (4 mm) in the fusion (PET and CT) image, in which SUV in each region was at least 1.5 and region localization was based on CT (Hounsfield units: −10 to −180).

### Statistical analyses

For analyses, the last 20 min of thermoneutral condition were averaged, for mild cold conditions a period of 30 min, starting 10 min after injection, was averaged. Percentage NST was calculated as the change in energy expenditure during mild cold conditions compared to a thermoneutral condition. Statistical analyses were performed using PASW Statistics 20.0 for Mac (SPSS). Pearson correlations were used to identify relationships between cold-induced body temperatures (temperatures at the end of cold stimulation) and BAT activity, and between the change in body temperatures (mild cold situation minus baseline) and BAT activity values. The effect of the cooling protocol was studied with a paired sample *T* test. Backward multiple linear regression analyses were performed to verify the contributions of other variables to NST and BAT activity. *P* < 0.05 was considered statistically significant.

## Results

### BAT activity and NST

In 94 % (34 of 36 BAT-positive) of the subjects, active BAT depots were found. Average BAT activity was 2.3 ± 0.7 SUV mean and 10.1 ± 6.6 SUV max. The cooling protocol significantly increased energy expenditure (thermoneutral: 5.0 ± 0.4, mild cold: 5.5 ± 0.5 kJ/min, *P* < 0.01). As a result, an average NST of 10.9 ± 7.3 % was found.

### Body temperatures

**Fig. 2:**
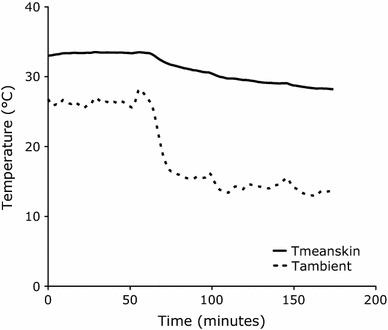
Visualization of mean skin (*T* mean skin; *solid line*) and ambient (*T* ambient; *dotted line*) temperatures during the measurements. The measurements started with a thermoneutral period of 60 min followed by an individualized cooling protocol

Upon cooling, mean skin temperature decreased from 33.1 ± 0.6 °C to 29.3 ± 0.8 °C (*P* < 0.01). In Fig. 2 mean skin and ambient temperature of a representative subject during the cooling protocol are visualized. Additionally, a significant drop in supraclavicular skin temperature was found (thermoneutral: 35.3 ± 0.5 °C; mild cold: 34.5 ± 0.7 °C, *P* < 0.01). Interestingly, supraclavicular skin temperature did not decrease to the same extent as mean skin temperature (−0.9 ± 0.6 drop in supraclavicular skin temperature and −3.8 ± 0.6 drop in mean skin temperature, *P* < 0.01). All but proximal and core temperatures showed a greater drop than supraclavicular skin temperature upon the cooling protocol (all *P* values < 0.01, Table 2). Highly interesting in this respect is the greater drop in skin temperatures of skin sites surrounding the supraclavicular area (head: −1.8 ± 0.6, chest: −2.2 ± 1.0 and deltoid: −4.0 ± 1.0 °C, all *P* values <0.01) compared to the drop in supraclavicular skin temperature (Table 2).

**Table 2:** Body temperatures during thermoneutral and mild cold conditions during the cooling protocol

	Air-cooling
Mean skin (°)	
Thermoneutral	33.1 ± 0.6
Mild cold	29.3 ± 0.8^a^
Change upon cold stimulation	−3.8 ± 0.6^b^
Core (°)	
Thermoneutral	36.8 ± 0.2
Mild cold	36.9 ± 0.3
Change upon cold stimulation	0.02 ± 0.22
Gradient underarm-fingertip (°)	
Thermoneutral	−0.10 ± 1.8
Mild cold	7.6 ± 2.4^a^
Change upon cold stimulation	7.9 ± 2.9^b^
Distal (°)	
Thermoneutral	30.8 ± 1.2
Mild cold	22.3 ± 1.3^a^
Change upon cold stimulation	−8.5 ± 1.2^b^
Proximal	
Thermoneutral	33.8 ± 1.2
Mild cold	31.6 ± 1.1^a^
Change upon cold stimulation	−2.2 ± 0.7
Gradient core-mean skin (°)	
Thermoneutral	3.0 ± 1.4
Mild cold	7.6 ± 0.8^a^
Change upon cold stimulation	3.8 ± 0.5^b^
Forehead (°)	
Thermoneutral	35.0 ± 0.6
Mild cold	33.2 ± 0.8^a^
Change upon cold stimulation	−1.8 ± 0.6^b^
Left upper chest (°)	
Thermoneutral	34.2 ± 1.8
Mild cold	32.0 ± 1.4^a^
Change upon cold stimulation	2.2 ± 1.0^b^
Left upper arm (deltoid muscle) (°)	
Thermoneutral	33.4 ± 0.9
Mild cold	29.4 ± 1.5^a^
Change upon cold stimulation	−4.0 ± 1.0^b^
Supraclavicular (°)	
Thermoneutral	35.3 ± 0.5
Mild cold	34.5 ± 0.7^a^
Change upon cold stimulation	−0.9 ± 0.6

Values are expressed as mean ± SD

Paired samples *t* test was used to test the effect of the cooling protocol and to test the difference with the change in supraclavicular skin temperature

^a^ *P* < 0.01 mild cold vs. thermoneutral; ^b^ *P* < 0.01 supraclavicular vs. other skin temperatures

### Correlations

**Fig. 3:**
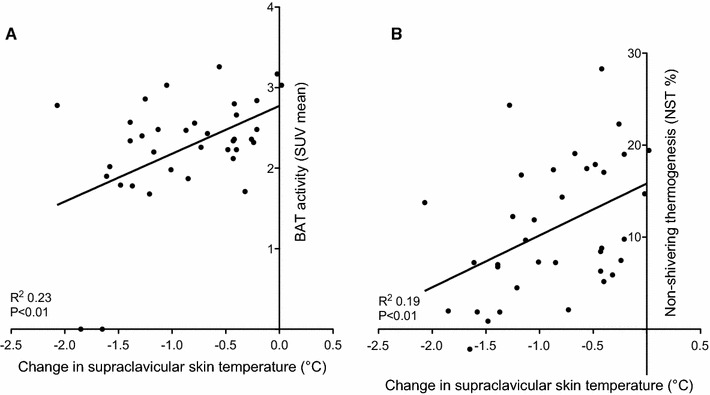
Correlations with change in supraclavicular skin temperature. **a** Correlation between change in supraclavicular skin temperature and BAT activity (expressed in SUV mean), **b** Correlation between change in supraclavicular skin temperature and NST (%)

Significant correlations were found between BAT activity and NST (*R* ^2^ = 0.40; *P* < 0.01). Correlation analyses showed a significant positive relation between the change in temperature at the supraclavicular site and SUV mean (*R* ^2^ = 0.23; *P* < 0.01) (Fig. 3a). Thus, subjects with a high BAT activity have a relatively small decrease in supraclavicular skin temperatures. No correlations were found between change in supraclavicular skin temperature with SUV max, and for cold-induced skin temperatures. Multiple linear regression analyses revealed no other significant contributors to BAT activity than the change in supraclavicular skin temperature and NST. Additionally a significant correlation was found between NST and the change in supraclavicular skin temperature (*R* ^2^ = 0.18; *P* < 0.01) (Fig. 3b). Multiple linear regression analyses revealed no other significant contributors to NST than the change in supraclavicular skin temperature and BAT activity.

## Discussion

In this study we showed a diminished decrease (−0.9 ± 0.6 °C) in supraclavicular skin temperature compared to the drop in mean skin temperature (−3.8 ± 0.6) and most other measured skin temperatures (Table 2). Additionally we found significant positive correlations between the change in supraclavicular skin temperature with NST and BAT activity. Subjects with a high NST and a high BAT activity have a relatively small decrease in supraclavicular skin temperature.

Several studies show that cold-induced BAT activity is related to NST. This indicates that BAT is important in maintaining core body temperatures upon cold exposure, by producing metabolic heat. Since the largest BAT depot in human adults is located above the clavicula (i.e., supraclavicular), skin overlaying this depot is likely to be affected by BAT activation. This study confirms the findings of Lee et al. [23] who showed a steeper decrease in skin temperature at the mediastinum (BAT-negative site) compared to the supraclavicular area (BAT-positive site) upon cold exposure (placement in a room of 17 °C for 30 min), whereby both sites were measured by infrared thermography. Thus, skin temperature measured by means of infrared thermography and iButtons (this study) provide similar results. On the other hand, Symonds et al. [30] showed an increased supraclavicular skin temperature upon a cold challenge. Their cooling protocol consisted of placement of feet or hands in water of 20 °C. The discrepancy in supraclavicular skin temperature may be explained by the different cooling protocol, i.e., local cooling instead of whole body cooling.

This study confirms earlier findings that BAT activity and NST are related [17, 26]. Subjects with high BAT activity values have a relatively large NST. These findings underscore the importance of BAT in maintaining core body temperatures. In this study, a significant correlation was found between the change in supraclavicular skin temperature and BAT activity. Subjects with a high BAT activity have a relatively small decrease in supraclavicular skin temperatures. The explained variance is relatively low (*R* ^2^ = 0.23); however, it indicates that the change in supraclavicular skin temperature gives an indication of BAT activity. Boon et al. [24] also studied supraclavicular skin temperature measured with iButtons in relation to BAT activity measured with [^18^F]FDG-PET/CT-imaging. In contrast to our findings, they reported a significant increase in supraclavicular skin temperature, whereas other skin temperatures significantly decreased upon cooling. Moreover, significant positive correlations were found between absolute cold-induced supraclavicular skin temperatures and total and clavicular BAT volume, and clavicular SUV max [24]. No correlation between the changes in supraclavicular skin temperatures and these variables was reported. The discrepancy between their results and ours might be explained by the differences in cooling protocol. Boon et al. [18] used water-cooling with a suit, while we used air-cooling in this study. Different cooling protocols result in different BAT activity values. Different BAT analyzing techniques were used which influences activity values. This could be another reason for discrepancy of the results.

The main function of BAT is the regulated production of heat in order to maintain core body temperatures. Clearly, this heat is not intended to increase skin temperature. In that perspective, most of the produced heat needs to be transported to core body sites. However, our results indicate that, most likely by means of perfusion or conduction, some of this heat ‘leaks’ towards the skin, resulting in relatively higher skin temperatures at sites covering BAT depots.

Several studies investigated (changes) in supraclavicular skin temperature upon a cold challenge using different cooling protocols. This study is the first using a strict temperature-controlled air-cooling protocol. It can be concluded that during cold stimulation the temperature decrease is less at skin overlaying supraclavicular BAT depots compared to other skin sites in human adults. Measuring changes in skin temperature could be useful to provide information on BAT activation upon repeated cold challenges. Additionally, this method could be used when information on BAT activity is needed in subjects that cannot undergo PET/CT-imaging. It should be taken into account that, due to a high inter-individual variation in the changes in supraclavicular skin temperature, this methodology cannot be used as a quantitative measure of BAT activity. The correlation between supraclavicular temperature BAT activity and NST indicates that changes in supraclavicular skin temperature can be used as a qualitative indicator of BAT activity and BAT thermogenesis.
